# Letter from Dr. Ruschenberger

**Published:** 1846-06

**Authors:** W. S. W. Ruschenberger

**Affiliations:** Surgeon U. S. Navy


*United States Naval Hospital,*
NEW YORK, May, 14th, 1846.

Prof. R. M. HUSTON,

SIR,—It is stated, page 289, of the Medical Examiner for May, 1846, that Phosphate of Ammonia "in the dose of only about three grains" produced a series of alarming symptoms, &c.

I have employed phosphate of ammonia in nine cases, in doses of ten grains repeated every four hours. In no case was the article used less than one week, and in several it was continued three, four, and even six weeks. The urine was tested before the medicine was prescribed and during its use; but no change in its constitution was detected by reagents, nor was there any change in the quantity or specific gravity of the secretion. In one case only did any amelioration of symptoms occur after the use of the phosphate of ammonia; whether this amelioration was *post* or *propter* I cannot decide; but, inasmuch as no perceptible effects whatever were produced in any one of the other eight cases, my present impression is that phosphate of ammonia is useless in the treatment of chronic rheumatism.

The article employed by me was procured from one of the most respectable druggists in Philadelphia.

Very respectfully, your ob't. serv't.,

W. S. W. RUSCHENBERGER, M. D.
Surgeon U. S. Navy.

